# Measuring agreement among experts in classifying camera images of similar species

**DOI:** 10.1002/ece3.4567

**Published:** 2018-10-30

**Authors:** TJ Gooliaff, Karen E. Hodges

**Affiliations:** ^1^ Department of Biology University of British Columbia Okanagan Kelowna British Columbia Canada

**Keywords:** bobcat, Canada lynx, expert identification, image classification, *Lynx canadensis*, *Lynx rufus*

## Abstract

Camera trapping and solicitation of wildlife images through citizen science have become common tools in ecological research. Such studies collect many wildlife images for which correct species classification is crucial; even low misclassification rates can result in erroneous estimation of the geographic range or habitat use of a species, potentially hindering conservation or management efforts. However, some species are difficult to tell apart, making species classification challenging—but the literature on classification agreement rates among experts remains sparse. Here, we measure agreement among experts in distinguishing between images of two similar congeneric species, bobcats (*Lynx rufus*) and Canada lynx (*Lynx canadensis*). We asked experts to classify the species in selected images to test whether the season, background habitat, time of day, and the visible features of each animal (e.g., face, legs, tail) affected agreement among experts about the species in each image. Overall, experts had moderate agreement (Fleiss’ kappa = 0.64), but experts had varying levels of agreement depending on these image characteristics. Most images (71%) had ≥1 expert classification of “unknown,” and many images (39%) had some experts classify the image as “bobcat” while others classified it as “lynx.” Further, experts were inconsistent even with themselves, changing their classifications of numerous images when they were asked to reclassify the same images months later. These results suggest that classification of images by a single expert is unreliable for similar‐looking species. Most of the images did obtain a clear majority classification from the experts, although we emphasize that even majority classifications may be incorrect. We recommend that researchers using wildlife images consult multiple species experts to increase confidence in their image classifications of similar sympatric species. Still, when the presence of a species with similar sympatrics must be conclusive, physical or genetic evidence should be required.

## 1 INTRODUCTION

Ecological research is experiencing an explosion in the use of wildlife imagery. Camera trapping has become a common noninvasive survey technique (Burton et al., 2015; O'Connell, Nichols, & Karanth, 2011; Rowcliffe & Carbone, 2008), especially for rare and elusive forest‐dwelling species (Furnas, Landers, Callas, & Matthews, 2017; Stewart et al., 2016), and has been used to obtain crucial ecological information (Caravaggi et al., 2017). Landscape‐scale camera grids or transects are increasing across the globe (McShea, Forrester, Costello, He, & Kays, 2016), and such sampling may be used to monitor global biodiversity in the future (Rich et al., 2017; Steenweg et al., 2017). For example, the project Snapshot Wisconsin currently has over 1,000 registered volunteers maintaining over 1,200 remote cameras and has collected over 22 million images since it was established in 2016 (Wisconsin Department of Natural Resources, 2018). Similarly, numerous websites and mobile phone applications encourage people to submit wildlife images for the purpose of assessing species’ distributions. For example, the United Kingdom Mammal Tracker application allows the general public to submit geo‐located images of 39 wildlife species (Mammal Watch South East, 2018).

Such camera networks and image‐solicitation projects can collect substantial data across broad scales, but the data may be of limited utility because of the need to classify the animals that the images contain (He et al., 2016; Newey et al., 2015; Wearn & Glover‐Kapfer, 2017). Researchers are typically interested in classifying each animal to the species level and in many cases even to individuals (Rich et al., 2014; Weingarth et al., 2012). However, classifying images is difficult when they are blurry, taken in poor lighting, show only part of the animal, or when only one image is available for a given animal (Meek, Vernes, & Falzon, 2013).

Further, even high‐quality images may be difficult to classify if the species has similar sympatrics (McShea et al., 2016; Swanson, Kosmala, Lintott, & Packer, 2016; Yu et al., 2013), especially if classifiers have a bias toward one sympatric species over another, perhaps based on the location or background habitat of an image. For example, rare species can have higher false‐positive and false‐negative errors than common species (McKelvey, Aubry, & Schwartz, 2008; Swanson et al., 2016). Similar concerns have also been raised for classification of acoustic records for groups such as bats, cetaceans, amphibians, and birds (Chambert, Waddle, Miller, Walls, & Nichols, 2017). Correct species classification is crucial; even low misclassification rates can lead to significant over‐ or underestimation of the occupancy, habitat preferences, or distribution of a species (Costa, Foody, Jiménez, & Silva, 2015; Miller et al., 2011; Molinari‐Jobin et al., 2012; Royle & Link, 2006), which could hinder conservation efforts (McKelvey et al., 2008).

Camera‐trapping and image‐solicitation studies have used various methods for image classification; manual classification by the lead researchers, hired technicians, or volunteer students is most common, but crowdsourcing from the general public (Swanson et al., 2016; Wisconsin Department of Natural Resources, 2018) and automated classification by computer software (Hiby et al., 2009; Jiang et al., 2015) have also been used. In our experience and observations of studies where images are manually classified, most images are classified by only a single person, but the number of classifiers and their expertise are rarely reported. Despite the fact that even highly trained experts are not always correct (Alexander & Gese, 2018; Austen, Bindemann, Griffiths, & Roberts, 2016; Gibbon, Bindermann, & Roberts, 2015; Meek et al., 2013; Swanson et al., 2016), the accuracy of image classifications is rarely questioned.

Classification of images by a single person may be adequate when classifying high‐quality images of species that are distinctive, such as mountain goats (*Oreamnos americanus*), porcupines (*Erethizon dorsatum*), and snow leopards (*Panthera uncia*), but may be unreliable for sympatric species that are similar in size, shape, or coloration (Meek et al., 2013). Many species across the globe fall into this category such as bears, deer, lemurs, some mustelids, felids and antelopes, as well as many bats, raptors, and owls. Specific examples include grizzly bear (*Ursus arctos*) versus black bear (*Ursus americanus*), mule deer (*Odocoileus hemionus*) versus white‐tailed deer (*Odocoileus virginianus*), nyala (*Tragelaphus angasii*) versus greater kudu (*Tragelaphus strepsiceros*), and sharp‐shinned hawk (*Accipiter striatus*) versus Cooper's hawk (*Accipiter cooperii*).

Here, we use bobcats (*Lynx rufus*) and Canada lynx (*Lynx canadensis*; hereafter lynx) as a case study to measure agreement among experts in their classifications of images of similar sympatrics. Bobcats and lynx are congeneric felids similar in size and appearance that are sympatric across southern Canada and the northern United States (Gooliaff, Weir, & Hodges, 2018; Hansen, 2007; McKelvey, Aubry, & Ortega, 2000). Although bobcats and lynx look similar, they have slight anatomical differences (Hansen, 2007; Lewis, 2016). Lynx have larger paws, longer legs and have more of an arched back compared to the straighter profile of bobcats. Lynx have more pronounced facial ruffs and longer ear‐tufts, as well as shorter, solid black‐tipped tails, as opposed to the longer, black and white‐tipped tails of bobcats. Bobcats also have black heel marks that are absent on lynx, and usually have more brownish and spotted pelage compared to the gray‐silver pelage of lynx.

Bobcats are common and are legally harvested in both countries, but lynx are federally listed as threatened in the contiguous US (US Fish & Wildlife Service, 2000). Classification of felid images in the contiguous US thus has direct conservation implications for lynx; bobcats falsely classified as lynx could result in false occupancy or distribution maps, or protection of areas that are not in fact used by lynx, whereas lynx misclassified as bobcats could result in under‐protection.

## 2 MATERIALS AND METHODS

**Figure 1:**
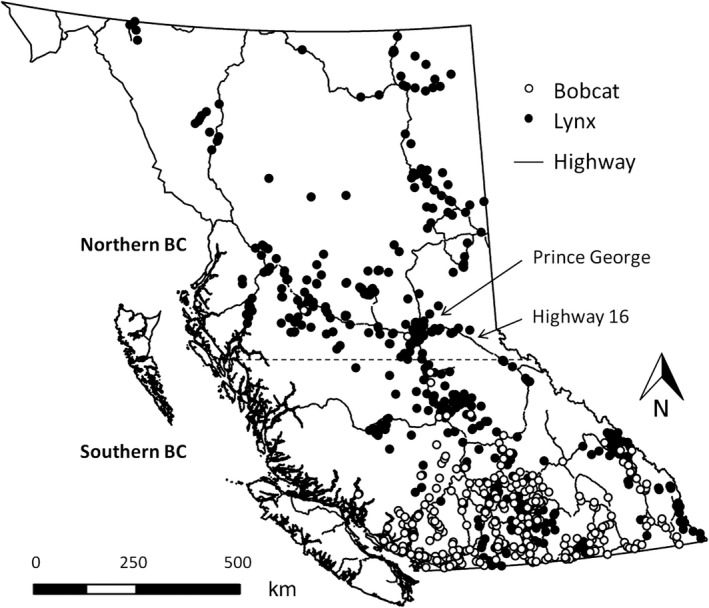
Images of bobcats (white circles; *n* = 805) and lynx (black circles; *n* = 807) taken during 2008–2017. These images were solicited from the public across British Columbia and here we map points based on our own classifications of the images. We also show our boundary between northern and southern BC (dotted line)

We measured agreement among experts in their classifications of bobcat and lynx images that we collected through citizen science. In a separate study, we solicited 4,399 images of bobcats and lynx from the public across British Columbia, Canada to examine the provincial distribution of each species (Figure 1; Gooliaff et al., 2018). We received 2,648 images (837 separate detections) of bobcats and lynx from remote cameras (*x̅* images per sequence = 3.2, median = 2, range = 1–38, 44% of detections had only a single image), and 1,736 images (748 separate detections) from conventional cameras and camera phones (*x̅* images per sequence = 2.3, median = 1, range = 1–26, 52% of detections had only a single image).

We subsampled those images to create six trials of images each designed as separate experiments to investigate different factors that we thought might affect agreement among the experts in their classifications of images; we tested the (a) season, (b) background habitat, (c) visible features of the animal, and (d) time of day in images, and (e) whether we provided the location of images to the experts (Table 1). The sixth trial was a retest of the first set of images, to assess whether experts were consistent in their classifications of the same images months later. We divided images into trials rather than providing them all at once both to make it easier for the experts and so that each factor that we tested was isolated in one set of images. Within each trial there were multiple categories of images (e.g., “summer” and “winter” categories in the “season” trial); we compared agreement among the experts in their classifications of the images between these different categories (Table 1).

**Table 1:** Characteristics of the 15 image categories within the six trials

Trial	Season	Background habitat	Time	Visible features	Location provided
1) Season					
Summer[Link] (*n* = 20)	Summer	Forest	Day	2 of: face, legs, or tail	No
Winter[Link] (*n* = 20)	Winter	Forest	Day	2 of: face, legs, or tail	No
2) Background habitat					
Forest (*n* = 20)	Summer	Forest	Day	2 of: face, legs, or tail	No
Grassland (*n* = 20)	Summer	Grassland	Day	2 of: face, legs, or tail	No
Developed[Link] (*n* = 20)	Summer	Developed	Day	2 of: face, legs, or tail	No
3) Visible features					
Full body (*n* = 20)	Winter	Forest	Day	Face, legs and tail	No
Face only (*n* = 20)	Winter	Forest	Day	Face only	No
Face and legs (*n* = 20)	Winter	Forest	Day	Face and legs only	No
Legs and tail[Link] (*n* = 19)	Winter	Forest	Day	Legs and tail only	No
4) Time					
Day (*n* = 20)	Winter	Forest	Day	2 of: face, legs, or tail	No
Night[Link] (*n* = 20)	Winter	Forest	Night	2 of: face, legs, or tail	No
5) Location					
a) Location provided					
Northern BC (*n* = 20)	Summer	Forest	Day	2 of: face, legs, or tail	Yes
Southern BC (*n* = 20)	Summer	Forest	Day	2 of: face, legs, or tail	Yes
b) Location not provided					
Northern BC (*n* = 20)	Summer	Forest	Day	2 of: face, legs, or tail	No
Southern BC (*n* = 20)	Summer	Forest	Day	2 of: face, legs, or tail	No
6) Consistency[Link]					

Images taken between April and September, and showing no snow.

Images taken between October and March, and showing snow.

Images showing human infrastructure, such as houses, barns, or patios.

One image was mistakenly included twice in this category; responses for the second time it appeared were removed from all analyses.

Black and white images taken at night.

This trial contained the same images as the first trial, but they were randomly reordered.

To select images for the different categories, we first chose images from the entire set that were of good photographic quality (i.e., the animal was in focus and not distant), were of single, alive, adult individuals that showed no bait or prey, and that were not submitted by any participating experts. We did not crop, edit or modify the images. We then randomly selected images to populate each category (Table 1). Within each category, all image characteristics (i.e., season, background habitat, visible features, and time of day) were consistent.

Each image was used only once, except for images in the “season” trial which were repeated as the “consistency” trial. We also mistakenly included one image twice in the “legs and tail” category. We disregarded the second classifications from the experts for this image in all analyses, which resulted in the “legs and tail” category containing 19 images rather than 20. Multiple images that were taken by the same remote camera, and thus that had the same background, were not included in the same trial. If the ratio of what we thought were bobcat and lynx images was below 4:1 for either species in any category, we randomly replaced images until at least that ratio was achieved, except for the “northern” images in the “location” trial because bobcats are likely absent in northern BC (Figure 1; Gooliaff et al., 2018). In total, we selected 299 images: 116 images (39%) from remote cameras and 183 images (61%) from conventional cameras.

We created weblinks for the six trials (Table 1) using FluidSurveys (http://www.fluidsurveys.com). We released trials online sequentially, two weeks apart, between January and April 2017. In each trial, experts were prompted to classify the species in each image by selecting “bobcat,” “lynx,” or “unknown.” The experts were not able to zoom in on images to ensure that experts based their classifications on the same view and detail of the images. The order of images in each trial was random, but was the same for all experts. Experts could not proceed to the next image without selecting an answer, and once selected, experts could not view previous images. However, experts were allowed to save unfinished trials and complete them at a later time. Trials were password protected, and we instructed experts to not consult with others; the experts did not know who else was participating in the experiment. Our study obtained ethics approval from the University of British Columbia (certificate # H16‐03169).

Experts were aware that we were measuring agreement among them in their classifications of images, but they were unaware of the conditions that we were testing in each trial. Experts were unaware of image locations to ensure that experts based their classifications on the images themselves and not on any contextual information. We provided the location for only half of the images in the "location" trial (Table 1), to test whether knowing such information affected agreement among the experts in their classifications of the images. These images were accompanied with a map of BC showing the location of the image with a red star. The map also included cities and highways to help orient the experts. Although location information would almost always be available for images collected in actual camera‐trapping or image‐solicitation studies, and thus, our experiment does not reflect a realistic scenario in this regard, we wanted to determine whether knowing such information might bias expert classification in these actual studies.

We selected 27 experts from across western North America to classify the images; we chose experts from (a) northern BC and the Yukon (*n* = 9), where lynx are common but bobcats are likely absent (Figure 1; Gooliaff et al., 2018), (b) southern BC (*n* = 8), where both species are common (Figure 1; Gooliaff et al., 2018), and (c) the northwestern contiguous US (*n* = 10), where lynx are rare but bobcats are common (Hansen, 2007; McKelvey et al., 2000). We considered people as bobcat or lynx experts if they were biologists who had field or image‐classification experience on either species. Even if somebody had experience working with only one species, we felt that they should be able to distinguish the species more familiar to them from the less‐familiar species. For example, if somebody had experience working with lynx but not bobcats, they should be able to tell that an image of a bobcat is “not a lynx.” All of the people who participated in our experiment agreed that they had relevant experience to be considered an expert. Our panel of experts represented people likely to participate in studies on one or both species, or who would likely be asked to classify bobcat or lynx images. The experts consisted of mesocarnivore and furbearer biologists from provincial, state, and federal government agencies, as well as private consultants and academics.

### 2.1 Statistical analysis

Our response variable was the number of experts that classified each image as “bobcat,” “lynx,” or “unknown.” Because we used images that were contributed by the public, we were unable to independently verify the species in each image and thus could not conclude whether expert classifications were accurate. Instead, we measured agreement among experts in their classifications of the images (hereafter agreement) using Fleiss’ kappa (*K*), which measures reliability among a group of classifiers. We calculated *K* using the R package *irr* (Gamer, Lemon, Fellows, & Singh, 2014) and calculated 95% confidence intervals based on 1,000 bootstrap iterations using the R package *boot* (Canty & Ripley, 2017). *K* is bound between −1 and 1; a value of 1 indicates perfect agreement, 0 indicates agreement that would occur by chance, and −1 indicates perfect disagreement (Fleiss, 1971).

*K* is commonly used in medical fields to measure agreement among clinicians in their diagnosis of certain conditions from images (Barnett, Glickman, Umorin, & Jalali, 2018; Farr, Guitton, & Ring, 2018; Vandenberk et al., 2018), but has also been used to measure agreement among biologists in identifying individual cougars (*Puma concolor*) from remote‐camera images (Alexander & Gese, 2018). There is no standardized method for interpreting or comparing *K* beyond relative differences between groups (Gwet, 2010). Many medical studies consider values >0.60 to represent “substantial” agreement (Landis & Koch, 1977); however, such studies often ask experts to rate the severity or progression of a disease or condition, whereas we asked experts to classify an animal species. Thus, in our study, we interpreted *K* more critically because experts were selecting from fewer and more distinct categories, conditions that typically increase *K* values (Sim & Wright, 2005).

We determined whether agreement varied between images with different characteristics (i.e., season, background habitat, visible features, and time of day) by comparing *K* between categories of images within each trial (Table 1). We also determined the combination of image characteristics that resulted in the highest and lowest agreement by pooling images with the same combination of characteristics from all categories. We determined whether knowing the location of an image affected agreement by comparing *K* when experts knew the location of an image to when they did not for images taken in northern and southern BC (Figure 1). We also determined whether agreement varied depending on the rarity of a species where experts lived by comparing *K* between experts from the three regions, and determined whether knowing the location of an image affected agreement within expert groups differently for either northern or southern images. We also determined whether experts were consistent in their classifications by having them unknowingly reclassify images from the first trial (“season” trial) 10 weeks later and calculating *K* between their first and second classifications of the same images.

In addition to calculating *K* across different kinds of images, we also calculated the proportion of agreement for individual images using the following equation, where *bobcat*,* lynx*, and *unknown* are the number of experts that classified an image as “bobcat,” “lynx,” and “unknown,” respectively, and *n* is the total number of experts:

$$\frac{{[(bobcat}^{2}+{\mathrm{lynx}}^{2}+{\mathrm{unknown}}^{2})-n]}{n\times (n-1)}$$

With three classification options, the proportion of agreement had an upper bound of 1.00, indicating perfect agreement and had a lower bound of 0.31, indicating perfect disagreement (i.e., of 27 experts, nine each classified an image as “bobcat,” “lynx,” and “unknown”).

Finally, we calculated the number of experts required to classify an image to reach a final classification (i.e., the number of experts at which the majority classification was unlikely to change by asking more experts). We calculated the mean probability that the majority classification (i.e., the classification of the greatest number of experts) of a randomly selected subset of one to 27 experts matched the majority classification of all 27 experts.

## 3 RESULTS

All 27 experts completed each of the six trials (Table 1). The following results refer to all images in the first five trials (*n* = 259 images); this set excludes the 40 images in the “location” trial for which we provided locations. The total number of individual expert classifications was 6,993 (27 experts × 259 images); the experts classified the images as “unknown” in 11% (*n* = 753) of classifications and as “bobcat” or “lynx” in 89% (*n* = 6,240) of classifications.

Of these 259 images, 71% (*n* = 185) had ≥1 experts classify that image as “unknown.” Experts reached a majority classification of “unknown” for 3% (*n* = 9) of images, but experts did not unanimously classify any images as “unknown.” Experts unanimously classified 24% (*n* = 61) of images as being either “bobcat” or “lynx,” while 39% (*n* = 101) of images had ≥1 experts classify that image as “bobcat” and ≥1 as “lynx.”

**Figure 2:**
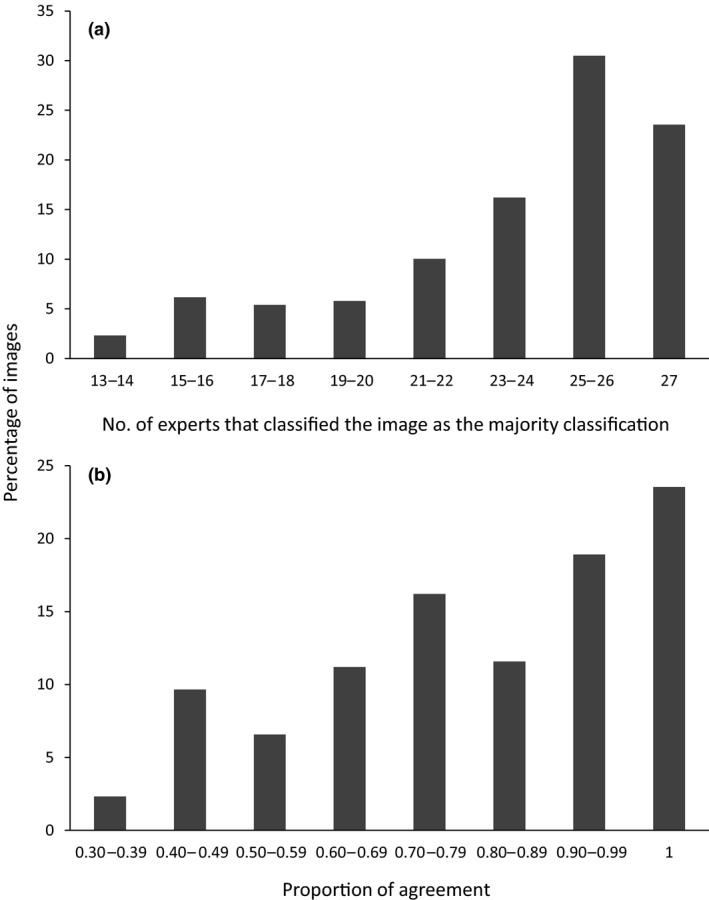
Distribution of (a) the number of experts that classified individual images as the majority classification and (b) the proportion of agreement scores among all 27 experts for individual images in all categories excluding the 40 images for which we provided locations (*n* = 259 images). With three classification options, the proportion of agreement had an upper bound of 1.00, indicating perfect agreement, and a lower bound of 0.31, indicating perfect disagreement

Overall, the 27 experts had moderate agreement in their classifications of the 259 images (*K* = 0.64, 95% CI = 0.60–0.68). The majority of images did not have a unanimous classification by the experts (76%; Figure 2a); the mean proportion of agreement score for individual images was 0.79 (*SD* = 0.19), but was highly variable (Figure 2b; Table 2). However, experts appeared to have similar agreement for each species; the mean proportion of agreement score was 0.84 (*SD* = 0.18, *n* = 92) and 0.77 (*SD* = 0.19, *n* = 167) for images that we had classified as “bobcat” and “lynx,” respectively.

**Table 2:** Examples of images with poor agreement among experts in their classifications (*n* = 27 experts). Images were cropped from original versions; thus, they do not show all of the background features observed by the experts that classified them. Images provided by: (A) Paul Morgan, (B) Amber Piva, (C) Jacqueline Brown, (D) Myrna Blake, (E) Bert Gregersen, (F) Scott MacDonald, (G) Donald Hendricks, and (H) John E. Marriott

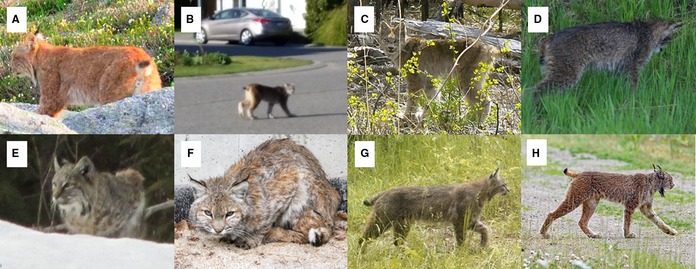

Image	No. of expert classifications			Proportion of agreement[Link]	Season	Background habitat	Visible features	Location
	Bobcat	Lynx	Unknown					
A	8	13	6	0.34	Summer	Grassland	Full body	McBride
B	7	15	5	0.39	Summer	Developed	Full body	Kamloops
C	6	16	5	0.41	Summer	Forest	Full body	Tatla Lake
D	8	3	16	0.43	Summer	Grassland	Legs and tail	Fort Nelson
E	5	5	17	0.44	Winter	Forest	Face only	Vernon
F	16	9	2	0.45	Summer	Developed	Full body	Invermere
G	17	7	3	0.46	Summer	Forest	Full body	Cache Creek
H	7	19	1	0.55	Summer	Grassland	Full body	Fort Nelson

The proportion of agreement had an upper bound of 1.00, indicating perfect agreement, and had a lower bound of 0.31, indicating perfect disagreement.

**Figure 3:**
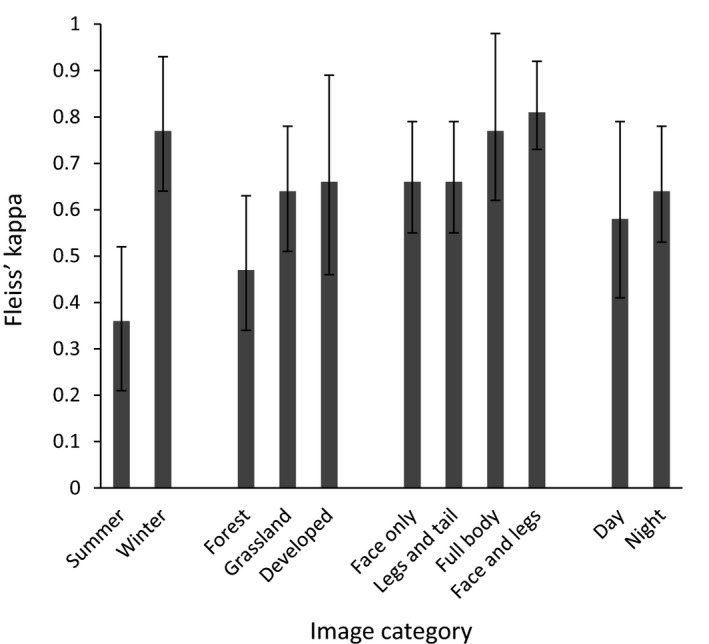
Agreement among all experts (*n* = 27) in their classifications of images within each category of images. All values of Fleiss’ kappa had a *p*‐value <0.001. Bars represent 95% confidence intervals. Fleiss’ kappa measures agreement among a group of classifiers; a value of 1 indicates perfect agreement, whereas a value of 0 indicates agreement that would occur by chance

Experts had varying levels of agreement between images with different characteristics (Table 3, Figure 3). Experts had far greater agreement for winter images than summer images. Experts had greater agreement for images with a background showing human infrastructure or grassland than for images with a forest background. Experts had greater agreement for images showing the full body or only the face and legs of an animal than images showing only the face or only the legs and tail of an animal. Experts had greater agreement for images taken at night than images taken during the day. Experts had the lowest agreement for daytime summer images with a forest background showing only the legs and tail of an animal (*K* = 0.34, 95% CI = 0.17–0.56, *n* = 15 images). Experts had the greatest agreement for daytime winter images with a forest background showing only the face and legs of an animal (*K* = 0.80, 95% CI = 0.72–0.88, *n* = 35 images). Experts had greater agreement when the location of an image was provided, and experts had greater agreement for southern images than northern images when they knew the location of an image (Table 3).

**Table 3:** Agreement among all experts (*n* = 27) in their classifications of images within each category of images. All values of Fleiss’ kappa had a *p*‐value <0.001

Category	No. of images	No. of images with a unanimous classification	Fleiss’ kappa (95% CI)[Link]
Season			
Summer	20	1	0.36 (0.21–0.52)
Winter	20	6	0.77 (0.64–0.93)
Background habitat			
Forest	20	3	0.47 (0.34–0.63)
Grassland	20	6	0.64 (0.51–0.78)
Developed	20	10	0.66 (0.46–0.89)
Visible features			
Face only	20	6	0.66 (0.55–0.79)
Legs and tail	19	3	0.66 (0.55–0.79)
Full body	20	6	0.77 (0.62–0.98)
Face and legs	20	8	0.81 (0.73–0.92)
Time			
Day	20	2	0.58 (0.41–0.79)
Night	20	4	0.64 (0.53–0.78)
Combinations (all daytime)[Link]			
Summer, forest, legs and tail	15	0	0.34 (0.17–0.56)
Summer, developed, full body	10	6	0.40 (0.18–0.77)
Summer, forest, full body	39	6	0.47 (0.35–0.60)
Summer, forest, face and legs	24	3	0.51 (0.37–0.69)
Summer, grassland, full body	12	4	0.58 (0.43–0.79)
Winter, forest, legs and tail	26	3	0.61 (0.50–0.75)
Winter, forest, face only	20	6	0.66 (0.55–0.80)
Winter, forest, full body	36	8	0.74 (0.65–0.85)
Winter, forest, face and legs	35	14	0.80 (0.72–0.88)
Location provided			
Northern BC	20	3	0.21 (0.08–0.38)
Southern BC	20	4	0.62 (0.45–0.83)
Total	40	7	0.50 (0.35–0.68)
Location not provided			
Northern BC	20	2	0.04 (0.01–0.07)
Southern BC	20	1	0.55 (0.44–0.69)
Total	40	3	0.44 (0.32–0.57)

Measures agreement among a group of classifiers; a value of 1 indicates perfect agreement, whereas a value of 0 indicates agreement that would occur by chance.

Images were pooled together from all categories excluding the 40 images for which we provided locations. Only combinations with ≥10 images are shown.

Experts were inconsistent even with themselves, as shown by comparing classifications of the 40 images in the “season” trial with classifications of those same images 10 weeks later. No expert had the same classifications for all images between the two trials; on average, experts changed their classifications on seven of the 40 images (*SD* = 3.4, range = 1–15). Experts had a mean consistency (i.e., agreement) with themselves of *K* = 0.67 (*SD* = 0.14, range = 0.29–0.94, *n* = 27). Further, we mistakenly included one image twice in the “legs and tail” category, and three experts changed their classification of this repeated image within the same trial. However, experts showed improved agreement between the first and last trials; whereas experts had an agreement of *K* = 0.55 (95% CI = 0.43–0.68) for images in the first trial (“season” trial), experts had an agreement of *K* = 0.63 (95% CI = 0.47–0.74) for the same images 10 weeks later (“consistency” trial).

**Figure 4:**
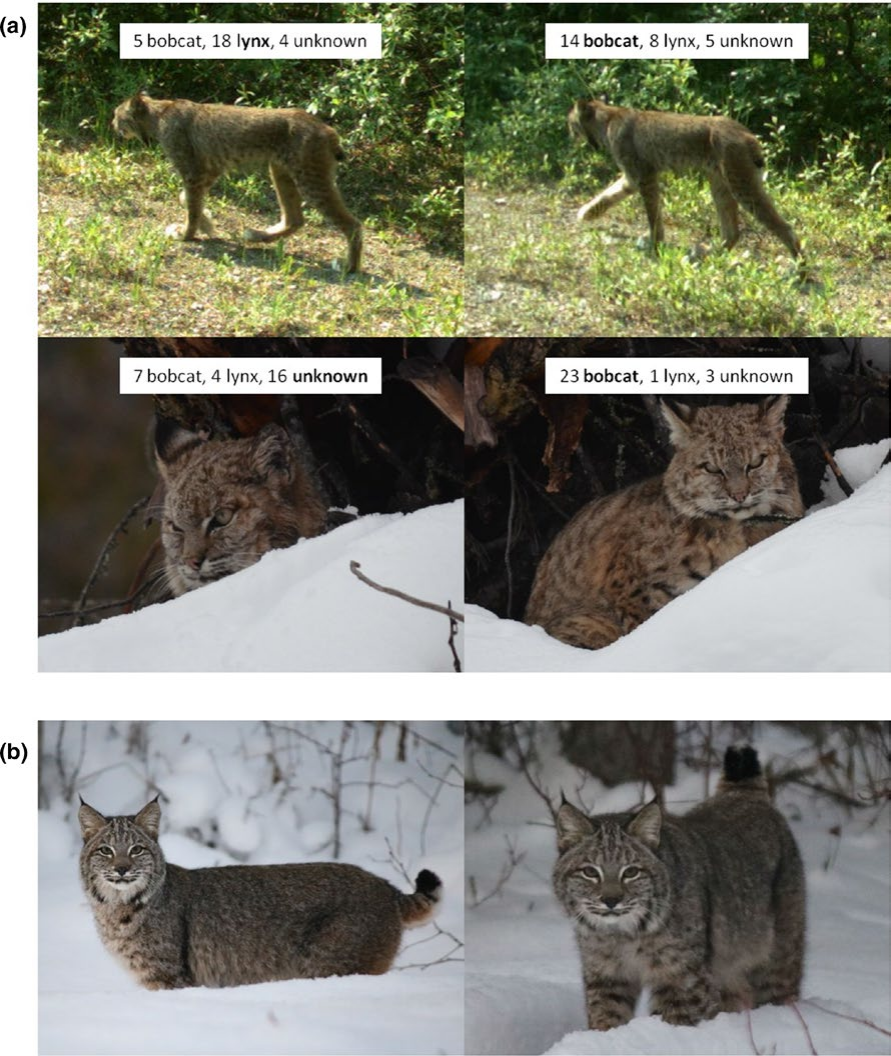
Examples of how the visible features of an animal and the location of an image can affect expert classification. (a) The top two images are of the same animal but show slightly varying body parts and had different majority classifications by the experts; the same occurred for the bottom two images. We show the number of experts that classified each image as “bobcat,” “lynx,” and “unknown.” (b) Both images are of the same animal taken near Prince George, British Columbia and have the same image characteristics. The image on the left was not included in our experiment but had a 4:4 split vote between bobcat and lynx among local biologists who were asked to classify the image. We included the image on the right in our experiment without providing its location; 26 experts classified the image as “bobcat”, and one expert classified the image as “unknown”. Images provided by (from top to bottom row): BC Parks, Emre Giffin, James Gagnon

Experts had contradictory majority classifications for different images of the same animal in two cases (Figure 4a). Out of all 299 images, there were 27 sets of images where this discrepancy could happen (i.e., where there were different images of the same animal but in different trials). The top two images in Figure 4a are of the same animal, but experts had a majority classification of “lynx” for the left image and “bobcat” for the right image. Similarly, the bottom two images in Figure 4a are of the same animal, but experts had a majority classification of “unknown” for the left image and “bobcat” for the right image. In both cases, experts did not know where each image was taken, and images had the same characteristics, but the images varied slightly in the perspective of the animal.

Experts from the three regions had similar levels of agreement; nine northern BC and Yukon experts had an agreement of *K = *0.64 (95% CI = 0.60–0.69), eight southern BC experts had an agreement of *K = *0.60 (95% CI = 0.55–0.64), and 10 northwestern US experts had an agreement of *K = *0.67 (95% = 0.63–0.71). Expert groups had different majority classifications for only 6% (*n* = 15) of images: 13 where one or two groups had a majority classification of “bobcat” or “lynx” while the other group(s) had a majority classification of “unknown,” and two where different groups had a majority classification of “bobcat” and “lynx.” The three expert groups had similar levels of agreement for images for which we provided locations, and also had similar consistency for retested images.

**Figure 5:**
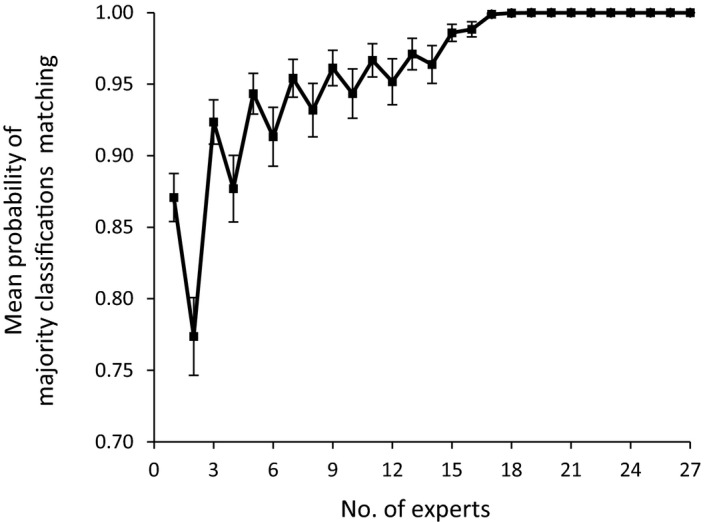
Mean probability that the majority classification of a randomly selected subset of experts matched the majority classification of all 27 experts, calculated across all images excluding the 40 images for which we provided locations (*n* = 259 images). Bars represent 95% confidence intervals. Probabilities are lower for even numbers of experts because of the likelihood of drawing a split vote, which is not possible for odd numbers of experts

Experts did reach a clear majority classification for most images (Figure 2a). On average, classifications of a single expert matched the majority classification of all 27 experts for 87% of the 259 images (median = 90%, range = 64%–97%). For five or more randomly selected experts, there was a mean probability of >0.90 that their majority classification matched the final majority classification of all experts, but a mean probability of 0.95 required 11 or more experts (Figure 5).

If the majority classification was correct for all images, then experts were incorrect in 4% of classifications, excluding classifications of “unknown” (i.e., 238 out of 6,240 individual expert classifications of either “bobcat” or “lynx” did not match the majority classification). If the majority classification was incorrect for all images, the misclassification rate would instead be 37%. The true misclassification rate is probably somewhere between these two bounds. Although we could not conclusively determine whether expert classifications were correct, 102 images were from locations where only one of the two species is known to be present: 29 bobcat images were from the southern coast of BC where lynx are likely absent, and 73 lynx images were from north of Highway 16 in northern BC where bobcats are likely absent (Figure 1; Gooliaff et al., 2018). If our classifications of these images are correct, then the majority classification of all experts was correct for all images in this subset excluding three images that had a majority classification of “unknown.” The misclassification rate for this subset of images would be 4%, excluding classifications of “unknown” (i.e., 91 out of 2,481 individual expert classifications of either “bobcat” or “lynx” did not match our classification).

Finally, we note that the two of us as authors had sequences for many and contextual information for all images and agreed with the majority classification of all 288 images in our experiment that had a majority classification of “bobcat” or “lynx” except for two; one majority classification by the experts was “lynx,” one “bobcat,” while we held the opposite views. One of those images is the top right image in Figure 4a.

## 4 DISCUSSION

We demonstrate far from perfect agreement among experts in distinguishing between images of two similar sympatric and congeneric species, bobcats and lynx. Previous work has shown that experts cannot reliably classify unique individuals from images for species that lack distinct markings (Alexander & Gese, 2018); we extend this unreliability to the species level for similar sympatrics. Experts had different levels of agreement for images with different characteristics, but in no case did experts have high enough agreement that we would consider the classification of such images by a single expert to be reliable. While we could not calculate the absolute misclassification rate because the true classifications of animals in the images were not independently confirmed, the misclassification rate was between 4% (if the majority classification was correct for all images) and 37% (if the majority classification was incorrect for all images).

Further, we were surprised at the frequent use of “unknown” as a classification by the experts in our study: experts classified images as “unknown” in 11% of classifications, and a striking 71% of images had ≥1 experts classify that image as “unknown.” Thus, in many cases, experts were not confident enough to classify the species in the image. These results are particularly troubling given that the images were all of high photographic quality. We do not know whether experts or novices would be more likely to classify images as “unknown”; experts may be aware of pitfalls in classification that novices do not know to look for, which could mean that experts use “unknown” more often than novices when images do not include critical defining features. Alternatively, novices may doubt their ability to classify a species, thus using “unknown” more frequently. Regardless, we provided the option of classifying each image as “unknown” rather than forcing experts to choose between “bobcat” and “lynx” to allow for such cases of genuine uncertainty. If we had forced experts to assign a species to each image, our calculated minimum misclassification rate of 4% would likely have been much higher. We recommend that researchers honor and trust cases of uncertainty where they cannot confidently classify the species in an image.

Expert agreement varied among different kinds of images. The largest difference was that experts had much lower agreement for summer images than for winter images. Bobcats and lynx are likely more difficult to distinguish in the summer because lynx have much lighter summer pelage and often become more brownish, and hence more similar to bobcats, whereas in the winter, lynx have thick, gray‐silver pelage. Experts also had lower agreement for images showing only the face or only the legs and tail than images showing the full body or only the face and legs of an animal, suggesting that it may be easiest to distinguish between the two species when both the face and legs are visible. Surprisingly, experts had slightly higher agreement for images taken at night than images taken during the day. Perhaps experts found it easier to distinguish between the two species at night because they were forced to focus on the physical features of each animal, rather than taking the color of an animal into account.

Expert agreement also depended on the background of images. Experts may have cued in on certain background features to aid in their classifications, for example, associating tree species or habitat with one species over the other. Some of the experts spontaneously commented to us after the study was complete that for some images they had based their classifications on the vegetation. Experts had lower agreement for images with a forest background than images with a background of grassland or human infrastructure, likely because grassland and developed habitats are more characteristic of bobcats, but both species use forests.

Further, we showed that the location of an image can also affect expert classification; experts had greater agreement when they were provided with the location of an image. Again, spontaneous post‐study comments from experts revealed that some experts used the location of an image to “confirm” their selections. However, while we expected experts to have greater agreement for images that we provided locations for, we were surprised to find that experts had greater agreement for southern images than northern images when the location was provided. We expected the opposite because bobcats are likely absent in northern BC; thus, there was essentially only one choice for northern images, whereas knowing the location of southern images should have provided little help since both species are common there (Figure 1; Gooliaff et al., 2018). Instead, some experts classified images from northern parts of the province as “bobcat,” counter to our expectation. This result suggests that those experts were not familiar with the distribution of bobcats in BC. Still, we strongly suspect that the location of an image can bias its classification if the person classifying the image has a preconceived idea of the species’ distribution, which can lead to misclassification of similar species if one species is thought to be extremely rare or absent in a particular area, when in fact it is present. As some species suffer range contractions and population declines, while others expand ranges with climate change, we think this possible location bias is worth further study.

For example, the left image in Figure 4b was taken near Prince George in 2016, and sent to us as part of our citizen science search for images (Gooliaff et al., 2018). At the time, there had never been a confirmed bobcat record that far north. We classified the image as “bobcat,” but the image was widely circulated on social media and the local news station, which sparked an intense debate among hunters, trappers, and naturalists as to whether the animal was a bobcat or lynx. Biologists in Prince George were asked by the local news station to classify the image, and initially four biologists thought “bobcat” and four biologists thought “lynx.” After additional images showing the animal's paws were shared, those biologists shifted toward classifying the image as “bobcat” or “possible hybrid” (K. Otter, University of Northern British Columbia, personal communication). The right image in Figure 4b is of the same animal and shares the same characteristics (i.e., season, background habitat, visible features, and time of day) as the left image. We asked experts to classify the right image in our experiment without providing its location; 26 experts classified the image as “bobcat” and one as “unknown.”

Despite the fact that experts unanimously classified only 24% (*n* = 61) of images, experts did reach a clear majority classification for most images. Thus, while classifications of an image by a single expert were unreliable, we believe that the final majority classifications were correct for most images. Our findings suggest that the location of an expert did not matter, as long as many experts were asked. We found only slight differences in agreement between experts from northern BC and the Yukon, southern BC, and the northwestern US, suggesting that experts were not biased by the rarity of a species in the area where they live.

### 4.1 Implications for studies using wildlife images

As photographic data become increasingly used in ecological studies for many groups of species (Rowcliffe & Carbone, 2008, Burton et al., 2015, Steenweg et al., 2017, Wisconsin Department of Natural Resources, 2018), we urge researchers to reevaluate and report how they classify their images. Reviews on the best practices for such studies focus on data management and sharing (Scotson et al., 2017; Wearn & Glover‐Kapfer, 2017); we highlight the need to also think carefully about image classification.

We acknowledge that our experiment does not mirror actual camera‐trapping or image‐solicitation studies because experts were provided with single independent images without any contextual information; however, we interpret our results to indicate that image classifications by a single expert are unreliable for species with similar sympatrics in such studies. In camera‐trapping studies, researchers know the landscape and local sites where cameras are installed and can measure distances from the camera to estimate animals’ sizes. In image‐solicitation studies, researchers know at least the general location of images. These design features might help with classification, but might also mean that the implicit bias of an individual classifier might result in misclassifications (e.g., the aforementioned case in Prince George where experts did not expect bobcats to be present). Both types of studies also often produce multiple images of the same animal, some of which show different body parts, which would likely improve classification. It would be valuable to conduct a study based on sequences of images for each animal to compare agreement among experts in their classifications when multiple rather than single images are available. However, we note that in our public solicitation for images (Gooliaff et al., 2018), approximately half of detections from remote cameras (44%) and conventional cameras (52%) still had only single images.

Although here we measured agreement among people in classifying single images, our experiment was based on the best‐case scenario of experts classifying images of high photographic quality. While we randomly selected images for our experiment to ensure that we did not consciously or subconsciously choose images that were easy or difficult to classify, our initial screen of using only high‐quality images meant that our collection of images was likely far easier to classify than images that would typically be collected in camera‐trapping or image‐solicitation studies, as blurry images or those with animals more distant from the camera would be more difficult to classify (Meek et al., 2013).

Misclassification rates would also likely be higher when images are classified by non‐experts, such as volunteers and crowdsourcing (McShea et al., 2016; Swanson et al., 2016; Wisconsin Department of Natural Resources, 2018). Image classifications by non‐experts may be suitable for species that are distinctive, but we strongly suggest caution when classifying images for species with similar sympatrics; we recommend that such images be flagged for classification by multiple experts. Specifically, we recommend that studies using wildlife images consult at least five species experts when classifying images showing species with similar sympatrics. Still, we stress that the majority classification of even five experts is not necessarily correct, only that the majority classification is unlikely to change by asking more experts.

Further, we recommend that researchers be explicit about their methods for classifying images. If researchers employ a design where most images are classified by one or two individuals who then consult with colleagues on difficult images, we urge such information to be provided, for example, specifying whether the main classifiers disagreed on the classification of such images, or whether all images that met a certain profile were flagged for further scrutiny. When sharing metadata from camera‐trapping and image‐solicitation studies, we recommend that researchers include information on the number of people who classified each image, whether those people were experts or non‐experts, and the individual classifications of each researcher. We also suggest that researchers make available the raw images to provide the option of reclassifying certain images in future studies, at least images that are highly influential to the final conclusions that are drawn (e.g., images from range edges, or images that are the only record of a species in a given locality).

Images have been described as being conclusive evidence for the presence of a species, even when that species is thought to be absent or extinct (McKelvey et al., 2008), but that is only true if the species in the image can be conclusively classified. We show that experts find it difficult to distinguish between images of similar species, which implies that images collected from camera trapping or public solicitation should not be taken as definitive evidence of species presence for any species that may be readily misclassified as a similar sympatric, such as bobcats and lynx, but rather as an initial subjective screen and then followed with definitive, objective survey methods such as noninvasive DNA sampling or live‐trapping.

## Conflict of interest

None declared.
